# Correlating Emotional Intelligence With Job Satisfaction: Evidence From a Cross-Sectional Study Among Secondary School Heads in Khyber Pakhtunkhwa, Pakistan

**DOI:** 10.3389/fpsyg.2020.00240

**Published:** 2020-03-13

**Authors:** Qaiser Suleman, Makhdoom Ali Syed, Ziarab Mahmood, Ishtiaq Hussain

**Affiliations:** ^1^Department of Education & Psychology, Kohat University of Science & Technology, Kohat, Pakistan; ^2^Department of Education, University of Kotli, Kotli, Pakistan; ^3^Department of Education, Mohi-ud-Din Islamic University, Nerian Sharif, Pakistan

**Keywords:** cross-sectional study, emotional intelligence, job satisfaction, relationship, secondary school heads

## Abstract

Emotional intelligence is extremely indispensable in functioning leadership positions as leaders wish everybody to fulfill his/her responsibilities and obligations effectively while job satisfaction has a direct association with the productivity and efficiency of an organization and also to individuals’ success. Therefore, this cross-sectional study examined the relationship between emotional intelligence and job satisfaction among secondary schools heads in Khyber Pakhtunkhwa. For this investigation, a total of 402 out of 884 secondary school heads were taken as a sample using a multistage sampling technique. The study was correlative, descriptive, and quantitative in nature, and survey research designed was used for collecting information from the participants. Statistical tools, i.e. mean, standard deviation, Pearson’s product-moment correlation, multiple linear regression, and analysis of variance, were applied. The findings showed that there was a moderate positive correlation between emotional intelligence and job satisfaction. Additionally, there was a moderate positive correlation between all the subdimensions of emotional intelligence and job satisfaction except emotional stability, where the correlation was also positive and the effect size weak. Furthermore, five dimensions of emotional intelligence such as managing relations, emotional stability, self-development, integrity, and altruistic behavior were found significant predictors of job satisfaction. Therefore, it is imperative to concentrate on those practices that promote emotional intelligence among secondary school heads.

## Introduction

Effective leadership is extremely indispensable for the accomplishment of organizational goals through effective management of material as well as human resources. Effective leadership has long been viewed as extremely essential for the outstanding performance of educational institutions by ensuring a vibrant, encouraging, and conducive environment; making available sufficient resources; and promoting good interpersonal relationships and improving students’ achievement (Marzano et al., 2005; Kythreotis et al., 2010). Successful leaders know their feelings, weaknesses, and strengths, and they possess a powerful sagacity of self-respect and self-esteem. Prolific leadership exhibits discipline, controls undesirable sentiments, maintains integrity, and shows flexibility. A school head is required to apply emotional and general intelligence to fulfill these obligations and commitment to ensure efficiently the mandates of a nation and also fulfill the mission of educational institutions effectively (Pashiardis, 2011). A successful leader can accomplish the organizational goals effectively, smooths a way to accomplish collective and individual goals, distributing and utilizing inadequate resources to fulfill the fundamental requirements and demands of the public through effective handling of resources. Leaders should have the capability to manage emotional situations effectively, to fabricate trust and understanding rapidly, to listen well, and to motivate the subordinates (Arinze, 2011). Therefore, effective leadership is extensively believed to be an essential organizational constituent and performing an outstanding contributory role in promoting individuals’ well-being as well as organizational efficiency. Leaders are incapable to execute their responsibilities efficiently provided they are knowledgeable, emotionally intelligent, satisfied, and safe in a workplace (Pellitteri, 2002). It clearly indicates that leaders with problems may contribute to many displeasing and disagreeable issues for the organization and its employees which damagingly influence the organizational performance on the whole. Therefore, emotional intelligence and job satisfaction of individuals are the most leading and dominant variables responsible for outstanding performance.

Emotional intelligence has been recognized to be the most important conceptualization that is progressively recognized in social psychology. Recently, emotional intelligence has been given much concentration in research especially in psychological research. It is regarded as one of the crucial elements of a successful life as well as psychological well-being (Bar-On, 2001). It was primarily explained by Salovey et al. (1990) who expressed that emotional intelligence is a competency to possess emotional knowledge, to perceive and control emotions well, and to stimulate intellectual and emotional growth. Afterward, the authors presented a revised and comprehensive description of emotional intelligence as the capability to observe feelings, coordinate feelings to encourage thoughts, and understand and control feelings to stimulate self-improvement (Mayer and Salovey, 1997). Mayer et al. (1990) were familiar with their incredible contribution to the rise of emotional intelligence. Afterward, in 1995, the development of emotional intelligence construct commenced with the work of Daniel Goleman having an incredible contribution. Goleman (1995) characterizes emotional intelligence as an assortment of skills or capabilities, i.e. having the capability to propel oneself and continue despite hindrances, to deal with impulse and dissatisfaction, to manage one’s mindsets, to keep sufferings from influencing the capability to think, to sympathize, and to be hopeful. Goleman’s methodology is perceived as a mixed model of emotional intelligence, which depicts a series of abilities and competencies comprising in five key areas: self-awareness, self-regulation, social skills, motivation, and empathy (Goleman, 1998). According to Bar-On (1997), emotional intelligence refers to a variety of non-intellectual competencies, capabilities, and skills which have an impact on one’s capability to do well with regard to the management of environmental demands and pressures. Bar-On (1997) classified emotional intelligence into five key parts, i.e. intrapersonal, adaptability, interpersonal, stress management, and general mood.

Emotional intelligence may be characterized as the capability to identify, persist, and control driving forces; communicate clearly; make incredible decisions; tackle issues; and perform with other individuals in such a way that makes companions and achievement (Stone et al., 1998). These competencies enable an individual to observe and control emotions, ensure poise and dignity, formulate objectives, promote empathy, ensure conflict resolutions, and promote competencies necessary for leadership and successful group participation (Elias, 2004). Bradberry and Greaves (2009) expressed that emotional intelligence is the individual’s ability, aptitude, recognition assignment, accurate appraisal, and management of his senses against other individuals and gatherings. The theories of emotional intelligence have been classified into two groups and models. The first group is known as the ability model describing that emotional intelligence is a particular sort of intellectual ability as well as a part of cognition intelligence. The second group is the mixed model in which the philosophers blend the abilities of intelligence with some personality attributes, for example, being optimistic. Emotional intelligence includes interpersonal intelligence and intrapersonal intelligence. Interpersonal intelligence is the external intelligence which an individual utilizes to understand and maintain relations with the other individuals. It is imperative for promoting characteristics like sympathy, empathy, and strengthening powerful relationships. On the other hand, intrapersonal intelligence is the internal intelligence that is used by an individual to understand himself which is necessary for self-awareness, self-inspiration, and self-regulation. The management of intrapersonal as well as interpersonal emotions is important for individuals’ academic and professional accomplishments. Individuals having a higher emotional intelligence are more expected to regulate, understand, and control emotions excellently in themselves as well as in the other individuals (Wijekoon et al., 2017).

Emotional intelligence is extremely indispensable in functioning leadership positions as leaders wish everybody to fulfill his/her responsibilities and obligations as brilliantly as would be prudent. Research reveals that emotional intelligence has significant influences on the leadership roles and success of employment, and it is the prime variable for a successful life that contributes to better individuals’ performance (Zijlmans et al., 2011). The leaders with an outstanding level of emotional intelligence apply their social capacities to move others, ensure durable relations with workers, and act as influential motivators by managing their emotions and perceiving their inadequacies (Chastukhina, 2012). Leaders with an outstanding degree of emotional intelligence may be increasingly able to achieve more productivity from less manpower. Emotionally intelligent leaders can unexpectedly make and promote emotionally intelligent teams due to social many-sided nature of the present-day organizations (Goleman, 2002).

Within the paradigm of emotional intelligence, three theories are considered the fundamentals of emotional intelligence (Goleman, 1995; Mayer and Salovey, 1997; Goleman, 1998; Bar-On, 2000; Mayer et al., 2000). These theories have been presented in the last decade as an endeavor to describe the capabilities, attributes, and skills related to emotional intelligence. Mayer and Salovey (1997) built up an emotional intelligence model concentrating on those areas that stimulate intelligence through the understanding of emotions. In their model, emotional intelligence determines the potential for achieving the proficiency of certain abilities in the field of emotional intelligence. Bar-On (2000) presented a trait model of emotional intelligence that gauges emotional intelligence through five domains, i.e. intrapersonal skills, interpersonal skills, adaptability, stress management, and general mood. Interpersonal skills include the management of relationships with other individuals. Intrapersonal skills stress on individuals’ concentration and commitment and also the capability to make planning and complete independent ventures. Stress management abilities involve a person’s capability to remain calm, use constructive managing strategies, and promote power supportive systems. Adaptability skills comprise of significant problem-solving aptitudes, flexibility, and the capability to reframe issues and their resolutions. The general mood is a pointer of hopefulness, optimism, and flexibility. Bar-On (1997) expressed that emotionally intelligent individuals are commonly optimistic, adaptable, realistic, and effective in resolving issues and facing stressful situations without losing control. Goleman’s model of emotional intelligence is a competency model that concentrates on the competencies of emotional intelligence that enable an individual to achieve accomplishment in the working place (Goleman, 1995; Goleman, 1998). Goleman’s mixed model demonstrates the facets of an individual’s personality in addition to the capability to stimulate oneself in social and emotional conditions (Goleman, 1995; Goleman, 1998). Likewise, Bar-On’s mixed model of emotional intelligence also indicates the role of interpersonal relationships on emotion, as well as skills endorsing adaptability and stress management (Bar-On and Parker, 2000). On the other hand, Mayer and Salovey’s skill model focuses on the abilities related to the processing of emotional information (Mayer and Salovey, 1997). Goleman (2002) found that leaders who utilize emotional intelligence skills may maintain loyalty and organizational productivity. Goleman (1998) additionally focused on emotional intelligence that comprises five parts: identifying one’s emotions (self-awareness), managing them, inspiring self, knowing emotions in other individuals (empathy), and maintaining relations.

Job satisfaction has a direct association with the productivity and efficiency of an organization and also to individuals’ success. It is the basic component that acts as a contributory factor to advancement, productivity, appreciation, income, development, and achievement, causing a feeling of fulfillment (Kaliski, 2007). It reflects the enthusiasm and gratification of an individual with his/her work. It is described as the feeling that is experienced at the end of accomplishing an assignment and might be desirable or undesirable reliant on the results of the task endeavored (Saiyadain, 2007). It is a many-sided and multifaceted phenomenon that portrays diverse things to different persons. It is generally associated with motivation, but the mode of association is not comprehensible. Satisfaction is different from motivation (Mullins, 2005). It is also described as a pleasant enthusiastic situation initiating from the occupational assessment, it represents a viable response to one’s profession as well as attitudes, the significant characteristics of employment gratification that are generally determined through organizations by means of the rating scale, workers’ responses (Kumari and Pandey, 2011).

Job satisfaction involves emotional, intellectual, and behavioral variables. The emotional variable refers to emotions with regard to employment, for example, exhaustion, tension, or pleasure. The cognitive or intellectual variable refers to beliefs as to one’s occupation, i.e. feeling that one’s profession is reasonably challenging and difficult. Lastly, the behavioral variable is comprised of employees’ practices related to their employment comprising of coming and remaining late, or appearing as sick, etc. Job satisfaction may affect capability, productivity, absenteeism, turnover, employees’ resignation, and finally employees’ prosperity (Usop et al., 2013). Dissatisfied individuals have a tendency to withdraw from organizations, whereas satisfied personnel are in good well-being and have a tendency to remain for a longer period in the organizations. Job satisfaction has various negative impacts such as despondency, uneasiness, and poor physiological and psychological prosperity influencing workers’ absenteeism, turnover, obligation, and commitment. Job satisfaction impacts individuals’ personal lives and therefore influences turnover and other essential business-related dispositions as well as demeanors. It acts as an outstanding turnover predictor and may influence learners’ judgment regarding the quality of services offered by the organization. Nevertheless, employees may be displeased with their occupation and ultimately, they have the intentions to leave the profession due to some reasons, for example, poor communication with contemporaries, high stress, lack of opportunities for advancement, and lack of recognition, etc. (Ucho et al., 2012).

In this technologically advanced era, every organization needs to accomplish outstanding achievement by means of productivity. Nevertheless, the accomplishment of this dream requires substantial satisfaction of workforces because they endeavor to increase more efforts to perform effectively to accomplish the stated goals. Likewise, the organizational achievement relies upon effective and creative individual execution (Kwateng et al., 2014). Currall et al. (2005) expressed that organizational profitability relies upon the execution of its workforces, and therefore, a high level of occupation fulfillment is essential for the outstanding execution of employees. Likewise, Meyer confirmed that job dissatisfaction unpleasantly affects the workers’ commitment which ultimately hampers the attainment of organizational goals and performance. Therefore, handsome and attractive packages are required for retaining higher performers (Meyer, 2014). Employment fulfillment helps in promising increasingly resourceful workforces and progressively organizational accomplishments. Those employees feeling satisfaction with their job are supposed to have outstanding excellence of work life when contrasted with those employees who are disappointed and their requirements are not fulfilled. Every employee in the working environment needs to perform a vital part for the progression and development of an organization and consequently, knowing employees’ occupational gratification is necessary for the change of execution and organizational efficiency (Nyanga et al., 2012).

According to Herzberg et al. (1959), there are five factors, namely, achievement, responsibility, work itself, recognition, and advancement, which act as strong predictors of job satisfaction. Other predictors are supervision, company policies, administration policies, compensation, working conditions, and interpersonal relationship. According to Lester (1987), the main components of job satisfaction are supervision, working conditions, colleagues, work itself, pay, responsibility, security, recognition, and advancement. Similarly, Sonmezer and Eryaman (2008) described that salary, advancement, ability utilization, social status, conducive working conditions, good relations, security, and creativity are the important elements of job satisfaction of the personnel of education. Treputtharat and Tayiam (2014) expressed that responsibility, performance standards, reward, unity, leadership, and success are the six components of the organizational setting which affect employees’ job satisfaction. According to Helms (2006), pay and money are the prime gorgeous factors of employees’ job satisfaction and motivation. Pay includes financial recognition for accomplishments. It is one of the tools to improve employee job satisfaction (Ketsela, 2017). Nyange (2013) expressed that advancement opportunities enable individuals to move toward advancement and growth which stimulate employees’ morale and inspire them to perform efficiently and more successfully. Subsequently, this builds organizational profitability and proficiency and stimulates the level of job satisfaction. Luthans (1998) affirmed that under favorable working conditions, e.g. a clean and fascinating environment, employees will perform their job effectively and successfully. Conversely, within an unpleasant working environment, like a hot and noisy environment, employees will not complete their work and subsequently experience dissatisfaction. Carrell et al. (1998) expressed that satisfaction is encouraged under effective supervision and the employees perceive their supervisor as sympathetic, cooperative, capable, and successful. Ineffective supervision comprises discriminating treatment of the supervisor and inability to correspond to workers’ problems, which thusly contribute to job dissatisfaction (Chung, 1977). Waqas et al. (2014) concluded that recognition, reward, and workplace environment are powerful influential factors affecting job satisfaction. But on the contrary, the involvement in the decision-making process has an insignificant association with job satisfaction.

Various research studies have been conducted to evaluate the association between emotional intelligence and job satisfaction locally and globally in various settings. After going through the findings of these studies, it has come to light that there is a substantial association between emotional intelligence and job satisfaction. Khanzada et al. (2018) found that there was a substantial positive relationship between emotional intelligence and job performance of the employees. The mediation outcomes revealed that job satisfaction moderately mediates between employees’ emotional intelligence and their job performance and reinforced the relationship. Rahman and Haleem (2018) found that emotional intelligence had a considerable positive influence on job satisfaction. Similarly, Khan et al. (2017) concluded that all the dimensions of emotional intelligence significantly predict job satisfaction. Additionally, their results indicated that among the indicators, self-assessment was found to be the most powerful predictor, whereas optimism was found to be the weakest predictor of job satisfaction. Naz and Liaquat (2015) found that emotional intelligence significantly impacts on employees’ job satisfaction and psychological ownership. Income level substantially impacts job satisfaction and psychological ownership positively. Conversely, it does not impact the emotional intelligence of the employees. Ashraf et al. (2014) found that there was sufficient evidence of a substantial association between emotional intelligence and job satisfaction with marital status as well as employment experience affecting it significantly. Hussain et al. (2014) investigated the association among three different variables related to secondary school teachers, and these variables are emotional intelligence, job satisfaction, and organizational commitment. Moreover, the study investigated the influence of gender and age in determining these aspects among the sample teachers. The outcomes indicate that there was a significant positive relationship between the three variables, i.e. emotional intelligence, job satisfaction, and organizational commitment.

### Rationale of the Study

After going through the literature, it was realized that emotional intelligence and job satisfaction are the two fundamental components for organizational advancement as well as for the overall individual prosperity. An extensive body of research has investigated the connection between emotional intelligence and job satisfaction globally in different fields (Gunavathy and Ayswarya, 2011; Çekmecelioğlu et al., 2012; Ealias and George, 2012; Lee and Ok, 2012; Zakieh and Aminilari, 2013; Ghoreishi et al., 2014; Masrek et al., 2014; Alnidawy, 2015; El-Badawy and Magdy, 2015; Mardanpour and Makvandi, 2015; Tabatabaei and Farzadmehr, 2015; Yusoff et al., 2016; Khanzada et al., 2018; Rahman and Haleem, 2018). A literature review revealed that in Pakistan, some related research studies have been conducted on teaching workforces as well as employees of other departments (Ashraf et al., 2014; Hussain et al., 2014; Naz and Liaquat, 2015; Khan et al., 2017; Khanzada et al., 2018; Rahman and Haleem, 2018), but unfortunately, the heads of the secondary schools have been badly ignored in this connection which shows the negligence of the educational researchers in Pakistan especially in the province of Khyber Pakhtunkhwa. No research study on educational leaders at the secondary level has been conducted previously in the province of Khyber Pakhtunkhwa with these variables. The role of a head in leading an institution toward advancement has become increasingly multifaceted and complex, and heads with problems may contribute to various undesirable and pessimistic consequences for educational institutes, as well as teaching and non-teaching workforces which damagingly and pessimistically influence the overall performance of the educational institutions. Without a competent and satisfied head, an educational institution cannot succeed in getting the right direction toward success and prosperity. In addition, he cannot perform his duties and responsibilities effectively for the welfare and prosperity of the institution until he is intelligent emotionally, capable, contented, and secured in the workplace. Therefore, it is necessary to make his job more respected, attractive, satisfying, and compensated. So, in this technologically advanced era, it is the intense need to conduct research on leading workforces with respect to these variables to make the educational institutions more advanced and progressive for quality education.

### Study Hypotheses

The purpose of the study was to examine the relationship between emotional intelligence and job satisfaction among secondary school heads in Khyber Pakhtunkhwa; therefore, the following null hypotheses were tested to achieve the research outcomes:

*Hypothesis 1*. There is no significant correlation between emotional intelligence and job satisfaction among secondary school heads.

*Hypothesis 2*. There is no significant correlation between the subdimensions of emotional intelligence and job satisfaction among secondary school heads.

*Hypothesis 3*. Subdimensions of emotional intelligence have no significant contribution in predicting job satisfaction among secondary school heads.

### Conceptual Framework of the Study

**FIGURE 1:**
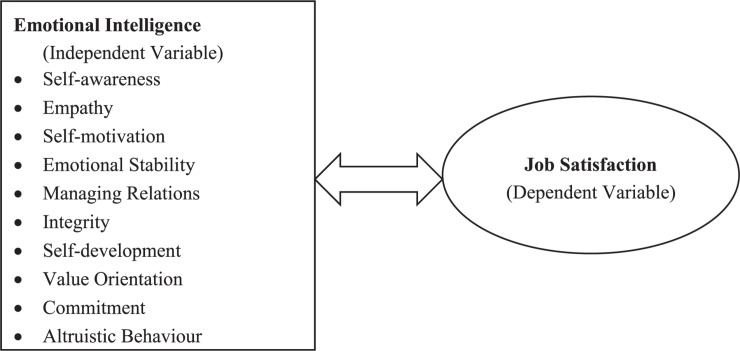
Conceptual Framework of the Study: Demonstrating the correlation model between the subdimensions of emotional intelligence and job satisfaction among secondary school heads.

A conceptual framework is the researcher’s idea which shows a proper direction in which the research study is going to be conducted. In this cross-sectional study, the conceptual framework is designed on the basis of Goleman’s Model and Herzberg Two-Factor Theory (Figure 1). According to Goleman (2001), emotional intelligence is composed of four main constructs, i.e. self-awareness, self-management, social awareness, and relationship management. In this study, 10 subdimensions of emotional intelligence (Figure 1) derived from the Goleman Model were used (Goleman, 2001). Herzberg et al. (1959) presented the two-factor theory and expressed that certain factors that cause employees’ job satisfaction are called motivators or satisfiers (i.e. achievement, recognition, career advancement, level of responsibility, etc.) while some other factors that lead to employees’ job dissatisfaction are called hygiene factors or dissatisfiers (i.e. organizational policies, salary, supervision, job security, working conditions, etc.) (Thomas, 2004).

## Materials and Methods

### Participants

This investigation was performed in the province Khyber Pakhtunkhwa which is situated in the northwestern region of Pakistan. It has been partitioned into seven divisions (such as Bannu Division, Kohat Division, Dera Ismail Khan Division, Hazara Division, Peshawar Division, Mardan Division, and Malakand Division) and 25 districts. Peshawar is the biggest metropolitan as well as the capital city of Khyber Pakhtunkhwa province. In the past, it was documented as North-West Frontier Province (NWFP). This investigation was done in 10 out of 25 districts because of financial constraints. In research, it is vital to confirm a precise representation of the population in terms of elements or subjects under examination, i.e. people, objects, associations, and so forth. In this investigation, all the secondary school heads in Khyber Pakhtunkhwa established the study population. There were a total of 2,108 secondary schools in the said province of Pakistan. In these schools, there were a total of 2,108 heads (male, *n* = 1,386; female, *n* = 722) (Table 1; EMIS, 2015).

**TABLE 1:** Population and sample size.

	Schools				Heads			
	Total		Sample		Total		Sample	
Districts	Male	Female	Male	Female	Male	Female	Male	Female
Nowshera	66	29	40	17	40	17	30	13
Kohat	47	27	28	16	28	16	21	12
Karak	56	26	37	16	37	16	28	12
Peshawar	85	55	51	33	51	33	38	25
Hangu	26	09	16	05	16	05	12	04
Charssadda	61	33	37	20	37	20	28	15
Abbottabad	69	45	41	27	41	27	31	20
Malakand	45	29	27	17	27	17	20	13
Bannu	59	40	35	24	35	24	26	18
Lakki Marwat	56	21	34	13	34	13	26	10
Total	570	314	346	188	346	188	260	142

**FIGURE 2:**
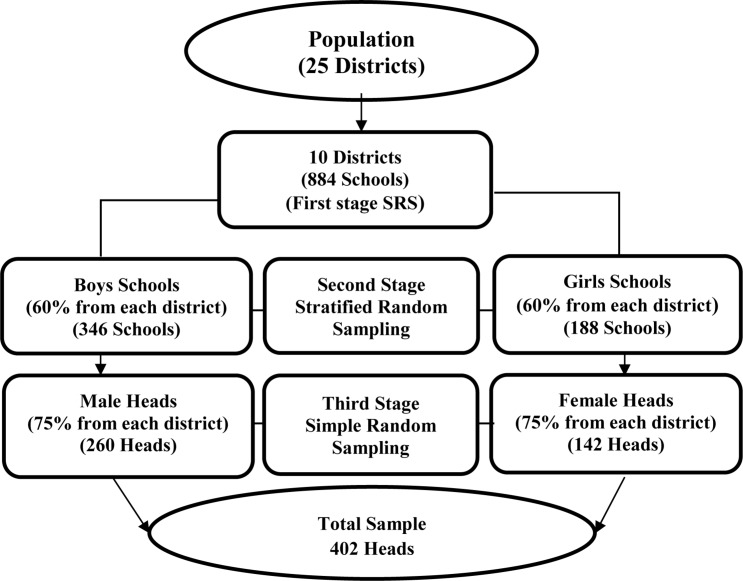
Multistage Sampling Model: Indicating the diagrammatical presentation of drawing the sample from the population. At the first stage, 10 out of 25 districts were selected through simple random sampling (SRS) technique. At the second stage, 60% Boys and 60% Girls Secondary Schools were selected through a stratified random sampling technique, and at the third stage, 75% male and 75% female secondary school heads were selected through SRS technique from each stratum.

In educational research, a multistage sampling procedure is broadly used worldwide because it is more precise, systematic, convenient, and reliable. In this cross-sectional study, a multistage sampling technique was used because the population was widely scattered. So, at the first stage, 10 out of 25 districts, i.e. Karak, Kohat, Abbottabad, Peshawar, Bannu, Nowshera, Lakki Marwat, Malakand, Charssada, and Hangu, were selected through a simple random sampling technique. At the second stage, 346 (60%) boys’ and 188 (60%) girls’ secondary schools were taken through a stratified sampling technique because the population was heterogeneous due to gender. In this way, two strata were made, i.e. boys’ schools and girls’ schools. At the third stage, 260 (75%) male and 142 (75%) female heads were chosen randomly from each stratum. In this manner, a sample of 402 secondary school heads (male, *n* = 260; female, *n* = 142) was chosen (Figure 2 and Table 1).

### Participants’ Demographic Characteristics

In order to conduct this cross-sectional study successfully, 402 participants joined after getting their permission. Among these participants, 260 were males and 142 were females. The demographic characteristics of the participants were analyzed on the basis of a simple percentage. Table 2 portrays that among the participants, 64.68% were males, and 35.32% were females. Regarding the age, only 6.71% of the participants were in the age group 30–34 years, 12.19% were grouped in 35–39 years, 18.90% were grouped in 40–44 years, and a high proportion of the participants (62.19%) were found having age 45 years and above. In terms of experience, 46.77% of the participants had 1–4 years’ experience, 25.62% had 5–9 years’ experience, 17.16% had 10–14 years’ experience, and 10.45% had 15 years and above of experience. With regard to academic qualification, 11.44% of participants were bachelor degree holders, 84.83% were master degree holders, 2.99% were M. Phil degree holders, and 0.75% were Ph.D. degree holders. Regarding professional qualification, 54.98% of the participants were bachelor degree holders, 41.79% were master degree holders, 2.74% were M. Phil degree holders, and 0.50% were Ph.D. degree holders. With respect to the locality, 22.39% of the participants belonged to urban localities, whereas 77.61% of the participants belonged to rural localities. In case of religion of the participants, 402 (100.0%) had Islamic religion, and no single participant was found having other religions than Islam.

**TABLE 2:** Participants’ demographic characteristics.

Characteristics	Categories	*n* (%)
Gender	Male	260 (64.68%)
	Female	142 (35.32%)
Age (in years)	30–34	27 (06.72%)
	35–39	49 (12.19%)
	40–44	76 (18.90%)
	45 and above	250 (62.19%)
Experience (in years)	01–04	188 (46.77%)
	05–09	103 (25.62%)
	10–14	69 (17.16%)
	15 and above	42 (10.45%)
Academic Qualification	BA/BSc	46 (11.44%)
	MA/MSc	341 (84.83%)
	MPhil	12 (02.99%)
	Ph.D.	3 (00.75%)
Professional Qualification	B. Ed	221 (54.98%)
	M. Ed	168 (41.79%)
	M. Phil (Edu)	11 (02.74%)
	Ph.D. (Edu)	02 (00.50%)
Locality	Urban	90 (22.39%)
	Rural	312 (77.61%)
Religion	Islam	402 (100.0%)
	Hinduism	00 (00.00%)
	Christianity	00 (00.00%)
	Others	00 (00.00%)

### Research Design and Measurements

In research, the research design is a methodical and systematic arrangement that helps the researchers to relate the theoretical framework, main research questions, gathering of information, and ways of statistical investigation to achieve precise and authentic outcomes (Yin, 2009). This cross-sectional study was correlative, quantitative, and descriptive. To accumulate the desired information from the participants, a survey research design was applied as the population was broadly disseminated, and it was difficult to accumulate information through other research instruments. The survey is commonly practiced for collecting quantifiable information from the respondents to gauge, perceive, assess, summarize, and generalize the research results and is perceived as an efficient and systematic way of accumulating data quantitatively (Zikmund, 2003). In the current investigation, two standardized instruments, Emotional Intelligence Scale (EIS) and Minnesota Satisfaction Questionnaire (MSQ), were utilized for gathering the required information. Both the instruments have been described as follows:

#### Emotional Intelligence Scale

In this cross-sectional study, the concept of emotional intelligence has been used which is proposed by Goleman. The EIS constructed by Hyde et al. (2002) was used for measuring emotional intelligence. This instrument has been designed in light of Goleman’s Model of emotional intelligence to assess the emotional intelligence of secondary school heads. Initially, Hyde et al. built up a scale comprising 106 items in total, but 34 items were observed to be extremely significant after performing statistical analysis, and the remaining items were omitted. The reliability was affirmed through a sample of 200 participants by ascertaining the split-half reliability coefficient and was found as 0.88. The content validity of the scale was computed as 0.93. In order to validate the scale, it was distributed among the Indian executives, and the desired information was accumulated. Based on factor analysis, 10 factors were found which comprise the sub-measurements of the emotional intelligence scale. The 10 sub-measurements of emotional intelligence are emotional stability, self-awareness, integrity, empathy, managing relations, commitment, self-motivation, value orientation, self-development, and altruistic behavior. *Self-awareness* is the idea that one exists as an individual, isolated from other individuals, with personal considerations. *Empathy* is the ability to recognize or understand the emotions and mental states of others. *Self-motivation* is the capability to inspire and stimulate one’s own self. *Emotional stability* is the capability of one’s character to keep stable in even unfavorable and stressful environments. *Managing relations* is the aptitude to inspire, motivate, encourage, influence, and develop others in order to achieve effective and successful outcomes. *Integrity* includes perceived regularity and uniformity of actions, beliefs, approaches, measures, and principles. *Self-development* is assuming individual liability and responsibility for one’s own learning and improvement through a process of appraisal, reflection, and taking action. *Value orientation* is the principles of good and bad that are acknowledged by an individual or a social gathering. *Commitment* intends to obligate or vow to something or somebody and can be alluded to personal duties and responsibilities. *Altruistic behavior* is being beneficial for other individuals. This scale was designed on five-point Likert scale, i.e. strongly agree to strongly disagree and was rated as 5 to 1 correspondingly (Jhaa and Singh, 2012).

#### Minnesota Satisfaction Questionnaire

The MSQ constructed by Weiss et al. (1977) is a well-known measuring tool used for gauging employees’ job satisfaction. The original version of the MSQ is composed of 20 dimensions divided into intrinsic and extrinsic facets, and each dimension is comprised of five questions. Intrinsic facets consist of 13 dimensions while extrinsic facets are comprised of seven areas such as achievement, ability utilization, authority, activity, coworkers, responsibility, moral values, independence, recognition, creativity, social status, social service, variety, school policies and practices, advancement, compensation, supervision (HR), security, supervision (technical), working condition. Based on cultural and societal background, slight modifications were made in the MSQ, and each dimension was confined to four questions. The MSQ has been constructed on a five-point Likert scale, i.e. very dissatisfied = 1, dissatisfied = 2, neither (neither satisfied nor dissatisfied) = 3, satisfied = 4, and very satisfied = 5.

### Pilot Testing

The pilot testing was performed to test the reliability and validity of the measuring instruments. It plays a contributing role in purifying the test and feasibility of the proposed study, to identify the possible issues with the proposed design, to refine the instrument, and to provide the investigators with a clear picture of the proposed study’s respondents, setting, and research methodology (Yusoff et al., 2016). A pilot study is carried out to explore weaknesses in research design and instruments and to ensure the provision of data for selecting a probability sample (Blumberg et al., 2005). Sekaran (2003) expressed that a pilot study facilitates a researcher in identifying problems and mistakes in the research instrument. He claims that it does not matter how many times a research instrument is revised, it is considered an operational document if it has been tested successfully in the field.

In this cross-sectional study, both the measuring instruments Emotional Intelligence Scale and Minnesota Satisfaction Questionnaire were standardized instruments having exceptional validity and reliability which are widely employed by scholars globally. Both the instruments were translated from English into Urdu language for a better understanding of the respondents. Therefore, it was imperative to confirm their validity. So, the pilot study was conducted in 25 government secondary schools selected from various districts of Khyber Pakhtunkhwa. In this way, the researchers distributed the instruments among 25 participants (males, *n* = 15; females, *n* = 10), and their responses were recorded. Based on the analysis, some minor language mistakes were found which were rectified. Conclusively, both the instruments were found appropriate for the current research study.

### Validity and Reliability

The validity and reliability are two basic components of a research study which should be kept in mind during proposing research design, statistical measurement, and assessment of the investigation (Patton, 2002). Validity is a basic key for viable and effective results of an investigation. Hence, without validating the research instruments, research is useless. Thus, validity is an essential condition for quantitative as well as qualitative research. The validity of quantitative data might be improved through precise sampling, reasonable research instrumentation, and appropriate analysis of data (Cohen et al., 2000). So, in addition to pilot testing, the validity and reliability were also confirmed in spite of the fact that these standardized instruments were exceedingly reliable and highly validated. The reason was their translation from English to Urdu language. So, the validity of EIS and MSQ was confirmed through five specialists having doctorate degrees and also having outstanding professionalism and experience.

With the aim to confirm the reliability of these standardized instruments, the internal consistency reliability procedure (Cronbach’s alpha reliability) was applied. After analysis, the average reliability coefficients of EIS and MSQ were found to be 0.86 and 0.86, respectively. The average internal consistency reliability of the subdimensions of the EIS shows that each dimension has a high reliability coefficient which confirms that EIS is a highly reliable research instrument (Table 3). Similarly, the average internal consistency reliability of the subdimensions of the MSQ indicates that each subdimension has a high reliability coefficient which proves that MSQ is an exceedingly reliable research instrument for data collection (Table 4).

**TABLE 3:** Reliability analysis of the emotional intelligence scale (EIS).

Subscales of EIS	No. of items	Cronbach’s alpha
Self-Awareness	4	0.897
Empathy	5	0.823
Emotional Stability	4	0.783
Self-Motivation	6	0.795
Managing Relations	4	0.813
Self-Development	2	0.896
Integrity	3	0.929
Commitment	2	0.836
Value Orientation	2	0.914
Altruistic Behavior	2	0.891
Mean	3.4	0.860

**TABLE 4:** Reliability analysis of the minnesota satisfaction questionnaire (MSQ).

Main divisions of MSQ	Subscales of MSQ	No. of items	Cronbach’s alpha
Intrinsic factors	Ability utilization	4	0.897
	Achievement	4	0.823
	Authority	4	0.783
	Activeness	4	0.795
	Creativity	4	0.929
	Coworkers	4	0.813
	Independence	4	0.896
	Moral values	4	0.914
	Social service	4	0.867
	Responsibility	4	0.917
	Recognition	4	0.836
	Social statues	4	0.764
	Variety	4	0.886
Extrinsic factors	Advancement	4	0.919
	Security	4	0.788
	Compensation	4	0.923
	Education policies and practices	4	0.837
	Supervision (Technical)	4	0.927
	Supervision (HR)	4	0.869
	Working condition	4	0.891
Mean		4	0.860

### Data Collection and Analysis

This cross-sectional study was formally approved by the Advanced Studies & Research Board (ASRB), Kohat University of Science & Technology (Pakistan), in its 39th meeting held on July 27, 2016. The main purpose of this approval was to discuss and confirm the various aspects of the study including the feasibility as well as the applicability of the research study, the significance of the study, the ethical aspects, and other related aspects. The process of data collection was initiated on September 15, 2016, and completed on February 15, 2017. Data from the participants of government high schools were collected through personal visits in four districts, namely, Karak, Kohat, Lakki Marwat, and Hangu. However, data were also gathered through mail from the participants serving in government high schools located in remote areas. That is why questionnaires were sent to participants on their institutional addresses in six districts, i.e. Abbottabad, Malakand, Charssadda, Peshawar, Nowshera, and Bannu. Respondents were furnished with a covering letter clarifying the aim of this investigation. They were told that their information will be kept top secret and would be utilized just for research purposes. Also, they were guaranteed that the information provided by them would be demolished after statistical analysis. In this way, after obtaining their informed consent, data were collected. Statistical tools such as mean, standard deviation, analysis of variance, Pearson’s correlation, and multiple linear regression were employed for statistical analysis of the information.

## Results

### Descriptive Analysis

#### Emotional Intelligence of Secondary School Heads

To analyze the emotional intelligence of the participants through descriptive statistics, different statistical tools were used such as mean, standard deviation, median, range, mode, skewness, and kurtosis. Table 5 portrays that secondary school heads were emotionally intelligent with nine subdimensions of emotional intelligence. The most rated dimension of emotional intelligence was altruistic behavior (*mean* = 3.67, *SD* = 0.797, *S*^2^ = 0.635) followed by self-awareness (*mean* = 3.58, *SD* = 0.682, *S*^2^ = 0.466) and self-motivation (*mean* = 3.57, *SD* = 0.616, *S*^2^ = 0.379). The other subdimensions of emotional intelligence were scored as integrity (*mean* = 3.54, *SD* = 0.717, *S*^2^ = 0.594), self-development (*mean* = 3.53, *SD* = 0.818, *S*^2^ = 0.669), value orientation (*mean* = 3.48, *SD* = 0.942, *S*^2^ = 0.887), managing relations (*mean* = 3.47, *SD* = 0.6896, *S*^2^ = 0.474), commitment (*mean* = 3.40, *SD* = 0.464, *S*^2^ = 0.215), and empathy (*mean* = 3.06, *SD* = 0.381, *S*^2^ = 0.464). In addition, it was revealed that secondary school heads were less emotionally intelligent regarding emotional stability (*mean* = 2.48, *SD* = 0.629, *S*^2^ = 0.395).

**TABLE 5:** Descriptive statistics of emotional intelligence among secondary school heads.

Variables	*N*	Min	Max	Mean ± SD	Range	Md	Mode	*S*^2^	SEM	Skewness		Kurtosis	
										Statistic	SE	Statistic	SE
SA	402	1.50	5.00	3.58 ± 0.682	3.50	3.50	3.50	0.466	0.0340	−0.096	0.122	−0.241	0.243
E	402	1.40	5.00	3.06 ± 0.681	3.60	3.00	3.00	0.464	0.0339	−0.145	0.122	−0.229	0.243
SM	402	1.67	5.00	3.57 ± 0.616	3.33	3.50	3.67	0.379	0.0307	−0.067	0.122	0.067	0.243
ES	402	1.00	5.00	2.46 ± 0.629	4.00	2.50	2.50	0.395	0.0314	0.389	0.122	0.354	0.243
MR	402	1.50	5.00	3.47 ± 0.689	3.50	3.50	3.50	0.474	0.0344	−0.137	0.122	−0.168	0.243
I	402	1.33	5.00	3.54 ± 0.771	3.67	3.67	3.33	0.594	0.0384	−0.347	0.122	−0.141	0.243
SDT	402	1.00	5.00	3.53 ± 0.818	4.00	3.50	4.00	0.669	0.0408	−0.243	0.122	−0.473	0.243
VO	402	1.00	5.00	3.48 ± 0.942	4.00	3.50	3.00	0.887	0.0470	−0.201	0.122	−0.575	0.243
C	402	2.31	4.78	3.40 ± 0.464	2.47	3.45	3.43	0.215	0.0231	−0.146	0.122	−0.129	0.243
AB	402	1.50	5.00	3.67 ± 0.797	3.50	3.14	3.50	0.635	0.0398	−0.326	0.122	−0.319	0.243

*Keys: Md, median; SD, standard deviation; S^2^, variance; SEM, standard error of the mean; SE, standard error; SA, Self-Awareness; SM, Self-Motivation; E, Empathy; MR, Managing Relations; ES, Emotional Stability; SDT, Self-Development; I, Integrity; C, Commitment; VO, Value Orientation; AB, Altruistic Behavior.*

#### Job Satisfaction of Secondary School Heads

In order to analyze the job satisfaction of the participants through descriptive statistics, various statistical tools such as mean, standard deviation, median, range, mode, skewness, and kurtosis were applied. As presented in Table 6, the descriptive statistics indicates that the secondary school heads showed satisfaction with their job position with respect to 12 subdimensions of job satisfaction. The most rated dimension was responsibility (*mean* = 3.74, *SD* = 0.693, *S*^2^ = 0.480) followed by social status (*mean* = 3.73, *SD* = 0.773, *S*^2^ = 0.597), independence (*mean* = 3.67, *SD* = 0.718, *S*^2^ = 0.515), security (*mean* = 3.67, *SD* = 0.761, *S*^2^ = 0.578), and social services (*mean* = 3.65, *SD* = *0.711*, *S*^2^ = 0.505). Other dimensions of job satisfaction were rated as achievement (*mean* = 3.62, *SD* = 0.762, *S*^2^ = 0.581), activity (*mean* = 3.62, *SD* = 0.772, *S*^2^ = 0.596), moral values (*mean* = 3.62, *SD* = 0.836, *S*^2^ = 0.700), coworkers (*mean* = 3.59, *SD* = 0.684, *S*^2^ = 0.469), authority (*mean* = 3.56, *SD* = 0.848, *S*^2^ = 0.719), variety (*mean* = 3.44, *SD* = 0.851, *S*^2^ = 0.725), and recognition (*mean* = 3.07, *SD* = 0.858, *S*^2^ = 0.736). Conversely, secondary school heads were dissatisfied with eight dimensions, i.e. ability utilization (*mean* = 2.32, *SD* = 0.833, *S*^2^ = 0.693), advancement (*mean* = 2.40, *SD* = 0.956, *S*^2^ = 0.914), school policies and practices (*mean* = 2.40, *SD* = 0.806, *S*^2^ = 0.650), compensation (*mean* = 2.33, *SD* = 0.790, *S*^2^ = *0.623*), creativity (*mean* = 2.70, *SD* = 0.913, *S*^2^ = 0.834), supervision (HR) (*mean* = 2.40, *SD* = 0.766, *S*^2^ = 0.587), supervision technical (*mean* = 2.44, *SD* = 0.890, *S*^2^ = 0.791), and working conditions (*mean* = 2.38, *SD* = 0.764, *S*^2^ = 0.584).

**TABLE 6:** Descriptive statistics of job satisfaction among secondary school heads.

Variables	*N*	Min	Max	Mean ± SD	*R*	Md	Mode	*S*^2^	SEM	Skewness		Kurtosis	
										Statistic	SE	Statistic	SE
Ability Utilization	402	1.00	5.00	2.32 ± 0.833	4.00	2.25	2.50	0.693	0.042	0.567	0.122	–0.023	0.243
Achievement	402	1.25	5.00	3.62 ± 0.762	3.75	3.50	4.00	0.581	0.038	–0.261	0.122	–0.144	0.243
Activity	402	1.50	5.00	3.62 ± 0.772	3.50	3.50	4.00	0.596	0.039	–0.216	0.122	–0.410	0.243
Advancement	402	1.00	5.00	2.40 ± 0.956	4.00	2.00	1.75	0.914	0.048	0.578	0.122	–0.736	0.243
Authority	402	1.25	5.00	3.56 ± 0.848	3.75	3.50	3.25	0.719	0.042	–0.232	0.122	–0.400	0.243
School Policies and Practices	402	1.00	5.00	2.40 ± 0.806	4.00	2.25	2.00	0.650	0.040	0.968	0.122	0.528	0.243
Compensation	402	1.00	5.00	2.33 ± 0.790	4.00	2.00	2.00	0.623	0.039	1.029	0.122	1.002	0.243
Coworkers	402	1.75	5.00	3.59 ± 0.684	3.25	3.50	3.25	0.469	0.034	–0.097	0.122	–0.336	0.243
Creativity	402	1.00	5.00	2.77 ± 0.913	4.00	2.75	2.00	0.834	0.046	0.329	0.122	–0.548	0.243
Independence	402	2.00	5.00	3.67 ± 0.718	3.00	3.75	3.25	0.515	0.036	–0.018	0.122	–0.600	0.243
Moral Values	402	1.00	5.00	3.62 ± 0.836	4.00	3.50	3.50	0.700	0.042	–0.218	0.122	–0.531	0.243
Recognition	402	1.00	5.00	3.07 ± 0.858	4.00	3.00	3.25	0.736	0.043	–0.030	0.122	–0.472	0.243
Responsibility	402	1.75	5.00	3.74 ± 0.693	3.25	3.75	4.00	0.480	0.035	–0.168	0.122	–0.315	0.243
Security	402	1.50	5.00	3.67 ± 0.761	3.50	3.75	4.00	0.578	0.038	–0.236	0.122	–0.376	0.243
Social Services	402	1.50	5.00	3.65 ± 0.711	3.50	3.75	3.50	0.505	0.035	–0.186	0.122	–0.202	0.243
Social Status	402	1.75	5.00	3.73 ± 0.773	3.25	3.75	4.00	0.597	0.039	–0.204	0.122	–0.626	0.243
Supervision (HR)	402	1.00	5.00	2.40 ± 0.766	4.00	2.25	2.00	0.587	0.038	0.789	0.122	0.281	0.243
Supervision (Tech)	402	1.00	5.00	2.44 ± 0.890	4.00	2.25	2.00	0.791	0.044	0.750	0.122	0.009	0.243
Variety	402	1.00	5.00	3.44 ± 0.851	4.00	2.50	3.50	0.725	0.043	–0.235	0.122	–0.294	0.243
Working Conditions	402	1.00	5.00	2.38 ± 0.764	4.00	2.25	2.00	0.584	0.038	0.723	0.122	0.293	0.243

*Keys: Md, median; SD, standard deviation; R, range; S^2^, variance; SEM, standard error of the mean; SE, standard error.*

### Inferential Analysis

#### Pearson’s Correlation Analysis

*Hypothesis 1*. There is no significant correlation between emotional intelligence and job satisfaction among secondary school heads.

To test the research hypothesis, as reflected in Table 7, a bivariate Pearson’s correlation was performed to examine the relationship between emotional intelligence and job satisfaction among secondary school heads. The value of r was calculated as 0.609 which undoubtedly shows a substantial (*p* < 0.01) positive relationship between emotional intelligence and job satisfaction. It plainly demonstrates that the higher the emotional intelligence of secondary school heads, then the higher will be their job satisfaction and vice versa. Therefore, the null hypothesis was rejected.

**TABLE 7:** Pearson’s product-moment correlation (*r*) between the emotional intelligence and job satisfaction among the heads of secondary schools.

Variables	Emotional intelligence	Job satisfaction
Emotional Intelligence	1.00	0.609**
Job Satisfaction	0.609**	1.00

**Correlation is significant at 0.01 level (two-tailed). Correlation Strength: 0.01 ≤ r ≤ 0.29 = Weak; 0.30 ≤ r ≤ 0.69 = Moderate; r ≥ 0.70 = Strong.

*Hypothesis 2*. There is no significant correlation between the subdimensions of emotional intelligence and job satisfaction among secondary school heads.

In order to test the research hypothesis, Pearson’s product-moment correlation was run between the subdimensions of emotional intelligence and job satisfaction among the heads of public secondary schools. Table 8 depicts that a moderate positive correlation was found between all the subdimensions of emotional intelligence and job satisfaction, i.e. emotional stability (*r* = 0.175), i.e. self-awareness (*r* = 0.388), empathy (*r* = 0.390), self-motivation (*r* = 0.450), managing relations (*r* = 0.470), integrity (*r* = 0.449), self-development (*r* = 0.343), value orientation (*r* = 0.386), commitment (*r* = 0.341), and altruistic behavior (*r* = 0.445). So, the research hypothesis was rejected. It means that emotionally intelligent heads will be satisfied with their employment.

**TABLE 8:** Pearson’s product-moment correlation analysis between the subdimensions of emotional intelligence and job satisfaction.

Variables	SA	E	SM	ES	MR	I	SDT	VO	C	AB	JS
SA	1.00										
E	0.391**	1.00									
SM	0.552**	0.452**	1.00								
ES	0.031	0.115*	0.099*	1.00							
MR	0.381**	0.477**	0.447**	–0.008	1.00						
I	0.592**	0.398**	0.663**	0.125*	0.364**	1.00					
SDT	0.283**	0.336**	0.442**	0.147**	0.323**	0.325**	1.00				
VO	0.420**	0.420**	0.406**	–0.037	0.507**	0.378**	0.260**	1.00			
C	0.411**	0.436**	0.409**	0.040	0.389**	0.432**	0.239**	0.448**	1.00		
AB	0.368**	0.321**	0.421**	0.058	0.406**	0.368**	0.190**	0.446**	0.350**	1.00	
JS	0.388**	0.390**	0.450**	0.175**	0.470**	0.449**	0.343**	0.386**	0.341**	0.445**	1.00

*Correlation is significant at 0.05 level (two-tailed), **Correlation is significant at 0.01 level (2-tailed). Correlation strength: 0.01 ≤ r ≤ 0.29 = Weak; 0.30 ≤ r ≤ 0.69 = Moderate; r ≥ 0.70 = Strong. Key: SA, Self-Awareness; SM, Self-Motivation; E, Empathy; MR, Managing Relations; ES, Emotional Stability; SDT, Self-Development; I, Integrity; C, Commitment; VO, Value Orientation; AB, Altruistic Behavior; JS, Job Satisfaction.

#### Multiple Linear Regression Analysis

*Hypothesis 3*. Subdimensions of emotional intelligence have no significant contribution in predicting job satisfaction among secondary school heads.

In order to test the hypothesis, different statistical tools, i.e. analysis of variance, collinearity, Durbin–Watson, and multiple linear regression were run to explore the role of each dimension of emotional intelligence as a predictor of job satisfaction. As shown in Table 9, the model is statistically significant because the value of analysis of variance was calculated as 25.267, which indicates that the result is significant (*p* < 0.05) statistically. Additionally, the table reflects that the value of R square is 0.393, which demonstrates that 39% of the variance in job satisfaction is substantially represented by the dimensions of emotional intelligence in the model. The regression analysis uncovered that among the dimensions of emotional intelligence, five dimensions were found to be substantial predictors and have a substantial positive influence on job satisfaction. Among these predictors, managing relations (*Beta* = 0.210) was investigated as the strongest predictor followed by altruistic behavior (*Beta* = 0.202), integrity (*Beta* = 0.154), emotional stability (*Beta* = 0.120), and self-development (*Beta* = 0.107) in defining job satisfaction positively. Conversely, self-awareness (*Beta* = 0.041), empathy (*Beta* = 0.055), self-motivation (*Beta* = 0.038), value orientation (*Beta* = 0.045), commitment (*Beta* = 0.015) have no substantial positive influence on job satisfaction. It clearly indicates that managing relations, altruistic behavior, integrity, emotional stability, and self-development are the substantial predictors that positively influence job satisfaction among heads of public secondary schools. Conclusively, the hypothesis was partially accepted.

**TABLE 9:** Multiple linear regression analysis to examine the role of independent variables (subdimensions of emotional intelligence) in predicting dependent variable (job satisfaction).

Model		Unstandardized coefficients		Standardized coefficients	*T*	*Sig.*	95% Confidence interval for B		Collinearity statistics		*R* Square	*F*	*Sig.*	Durbin–*Watson*
										
		*B*	SE	β			Lower	Upper	Tolerance	VIF				
Independent	(Constant)	1.649	0.116		14.271	0.000*	1.422	1.876						
variables	SA	0.019	0.025	0.041	0.780	0.436	–0.029	0.068	0.560	1.785				
	E	0.026	0.023	0.055	1.111	0.267	–0.020	0.072	0.630	1.587				
	SM	0.020	0.031	0.038	0.634	0.526	–0.041	0.081	0.438	2.284				
	ES	0.061	0.021	0.120*	2.955	0.003*	0.021	0.102	0.943	1.060				
	MR	0.098	0.024	0.210*	4.140	0.000*	0.052	0.145	0.601	1.663	0.393	25.27	0.00	1.38
	I	0.064	0.024	0.154*	2.665	0.008*	0.017	0.111	0.468	2.137				
	SDT	0.042	0.018	0.107*	2.367	0.018*	0.007	0.077	0.759	1.318				
	VO	0.015	0.017	0.045	0.884	0.377	–0.019	0.050	0.595	1.681				
	C	0.010	0.034	0.015	0.308	0.758	–0.056	0.076	0.662	1.510				
	AB	0.082	0.019	0.202*	4.283	0.000*	0.044	0.119	0.698	1.433				

*Significant predictors. Dependent variable: Job satisfaction. Independent Variables: SA, Self-Awareness; SM, Self-Motivation; E, Empathy; MR, Managing Relations; ES, Emotional Stability; SDT, Self-Development; I, Integrity; C, Commitment; VO, Value Orientation; AB, Altruistic Behavior.

## Discussion

In this technologically advanced era, every organization needs to accomplish outstanding achievements in terms of productivity and efficiency. Nevertheless, the accomplishment of this dream requires substantial satisfaction of workforces as they endeavor to increase more efforts to perform effectively to achieve the organizational goals. In this connection, emotional intelligence performs a substantial role in achieving organizational goals. The association between emotional intelligence and job satisfaction has caught the consideration of the investigators as emotional intelligence is playing a vital role in envisaging employees’ job satisfaction (Ghoreishi et al., 2014). Therefore, several research studies have been conducted to examine the association between emotional intelligence and job satisfaction (Anari, 2012; Çekmecelioğlu et al., 2012; Ealias and George, 2012; Lee and Ok, 2012; Zakieh and Aminilari, 2013). Likewise, this cross-sectional study also examined the relationship between emotional intelligence and job satisfaction. The results revealed that there is a moderate positive correlation between emotional intelligence and job satisfaction, which means that emotional intelligence is directly associated with job satisfaction. It is evident from this relationship that the higher the emotional intelligence of an individual, the higher will be his job satisfaction. Additionally, the results showed that there is a moderate correlation between all the subdimensions of emotional intelligence and job satisfaction, i.e. self-motivation, self-awareness, empathy, managing relations, emotional stability, integrity, value orientation, self-development, commitment, and altruistic behavior. It explicitly indicates that all these subdimensions of emotional intelligence have a substantial positive relationship with job satisfaction which shows that emotionally intelligent secondary school heads will perceive a higher level of job satisfaction. The findings of the study are consistent with the results of Anari (2012) who explored a significant positive correlation between emotional intelligence and job satisfaction. Likewise, Emdady and Bagheri (2013) found a substantial positive relationship between emotional intelligence and job satisfaction among workforces in Sama organization in Iran. According to Grund and Sliwka (2001), emotionally intelligent employees possess a higher level of job satisfaction because emotionally intelligent personnel can identify approaches to overcome the potential negative outcomes caused by stressful conditions. Conversely, those with less emotional intelligence are not capable to handle stressful situations effectively. The results are also supported by other previous research studies in which a substantial positive correlation between emotional intelligence and job satisfaction was reported (Lee and Ok, 2012; Mousavi et al., 2012; Trivellas et al., 2013; Alnidawy, 2015; Tagoe and Quarshie, 2016; Yusoff et al., 2016). It is evident from the findings that an individual having a high level of emotional intelligence will possess a high level of job satisfaction. On the other hand, surprisingly, Mandip et al. (2012), Ghoreishi et al. (2014), and El-Badawy and Magdy (2015) found no substantial association between emotional intelligence and job satisfaction.

In order to investigate the role of each subdimension of emotional intelligence as a predictor of job satisfaction, multiple linear regression analysis was performed. The outcomes showed that among the dimensions of emotional intelligence, five subdimensions were found substantial predictors of job satisfaction and have a considerable positive influence on job satisfaction, i.e. emotional stability, self-development, integrity, managing relations, and altruistic behavior. It plainly shows that job satisfaction will be positively affected by these subdimensions of emotional intelligence. On the contrary, self-awareness, empathy, self-motivation, value orientation, and commitment have no significant positive effect on job satisfaction. The findings of the study are in line to some extent with few research studies in which it was concluded that emotional intelligence predicts job satisfaction (Abraham, 2000; Livingstone, 2001; Thiebaut et al., 2005).

This study has some limitations. Firstly, only a quantitative research method has been used in this study. Therefore, a mixed-method research methodology, i.e. quantitative as well as a qualitative methodology may be used to investigate the same problem in future research. Secondly, the problem has been investigated through standardized tools, and there may be a slight difference in the findings if the problem may be investigated through self-developed measuring instruments. Thirdly, demographic variables, i.e. job experience, age, academic and professional qualification, locality, nature of the job, etc. may affect the results of the study, but these variables were not taken into consideration in this investigation. So, this limitation can be eliminated through a future research study by considering these demographic variables. Fourthly, this cross-sectional study was conducted in only 10 districts of Khyber Pakhtunkhwa. The results may differ to some extent if the same research study would be carried out in all districts of Khyber Pakhtunkhwa. So, the collection of data from all districts with a larger sample size will overcome this limitation.

## Conclusion

Emotional intelligence is a basic variable that ensures job satisfaction of individuals and hence stimulates the overall productivity of an organization. A moderate positive association was found between emotional intelligence and job satisfaction. Emotional intelligence predicts job satisfaction, and five dimensions, i.e. integrity, emotional stability, self-development, managing relations, and altruistic behavior, were found to be substantial predictors of job satisfaction. It clearly shows that emotional intelligence is directly related to job satisfaction; the higher the emotional intelligence, the higher will be the level of their job satisfaction.

## Recommendations

Emotional intelligence is closely linked with the efficiency and productivity of the workplace. Hence, it is imperative to emphasize those practices which subsidize to promote emotional intelligence and commitment among secondary school heads. In the recruitment process, preference should be given to those secondary school heads who are more emotionally intelligent. To boost up the level of emotional intelligence and job satisfaction of secondary school heads, workshops, seminars, and conferences should be held. As emotional stability, integrity, self-development, managing relations, and altruistic behavior proved to be the significant predictors of job satisfaction, therefore, secondary school heads should be developed with these dimensions of emotional intelligence through advanced professional training programs, seminars, and conferences. The curriculum of educational management and administration should be revised and modernized by including subjects on emotional intelligence and job satisfaction. Secondary school heads should be provided with handsome compensation and other incentives. The Ministry of Education should devise productive and effective education policies promising to the employees’ prosperity and organizational productivity. All the stakeholders should be taken into confidence during the process of policy formulation particularly schools’ heads and teachers for providing their valuable suggestions and experiences regarding school overall performance. Furthermore, necessary measures should be taken to implement education policies effectively. In addition, Elementary & Secondary Education Department Khyber Pakthunkhwa should have a collaboration with policy makers to formulate rewarding and comprehensive strategies for enhancing the level of job satisfaction of secondary school heads as well as making their job more attractive to stimulate their morale for yielding fruitful and productive outcomes. For future research studies, it is recommended that the same research study should be conducted at elementary, higher secondary, and tertiary levels in Khyber Pakhtunkhwa as well as in other provinces of Pakistan.

## Data Availability Statement

The datasets generated for this study are available on request to the corresponding author.

## Ethics Statement

The studies involving human participants were reviewed and approved by the Department of Education & Psychology as well as Advance Studies and Research Board (ASRB), Kohat University of Science & Technology, Pakistan. The participants provided their written informed consent to participate in this study.

## Author Contributions

QS wrote the original manuscript and contributed in conceptualization, introduction, investigation, methodology, and formal analysis. MS supervised the research work and contributed in methodology, reviewing and editing. ZM contributed in reviewing and editing, and methodology. IH supervised, reviewed, edited, and validated the research work.

## Conflict of Interest

The authors declare that the research was conducted in the absence of any commercial or financial relationships that could be construed as a potential conflict of interest.
